# Mobile-Based Application Interventions to Enhance Cancer Control and Care in Low- and Middle-Income Countries: A Systematic Review

**DOI:** 10.3389/ijph.2023.1606413

**Published:** 2023-12-05

**Authors:** Andrew Donkor, Jennifer Akyen Ayitey, Prince Nyansah Adotey, Esther Oparebea Ofori, Doris Kitson-Mills, Verna Vanderpuye, Samuel Yaw Opoku, Tim Luckett, Meera R. Agar, Penelope Engel-Hills

**Affiliations:** ^1^ Department of Medical Imaging, Kwame Nkrumah University of Science and Technology, Kumasi, Ghana; ^2^ College of Nursing and Health Sciences, Flinders University, Adelaide, SA, Australia; ^3^ Faculty of Health, University of Technology Sydney, Ultimo, NSW, Australia; ^4^ Korle Bu Teaching Hospital, Accra, Ghana; ^5^ Catholic University College of Ghana, Sunyani, Ghana; ^6^ Faculty of Health and Wellness Sciences, Cape Peninsula University of Technology, Cape Town, South Africa

**Keywords:** mobile applications, cancer screenings, prevention, early detection of cancer, palliative care

## Abstract

**Objective:** To identify and appraise mobile-based application (mAPP) interventions that have been used to support cancer control and care in low- and middle-income countries (LMICs).

**Methods:** Four electronic databases were systematically searched for studies that reported primary research findings related to mAPP interventions applied in oncology settings in LMICs. A narrative synthesis was performed using the Mhealth Index and Navigation Database as an analytical framework.

**Results:** Twenty studies reporting 18 cancer control and care mAPPs were included in this review. Among these mAPPs, ten focused on prevention, screening and early detection of cancer, five provided information to optimise supportive and palliative care, two provided support to assist treatment-shared decision-making and one covered information for follow-up and survivorship care.

**Conclusion:** Cancer mAPP interventions are gradually gaining attention in LMICs as they provide unique resources for empowering and strengthening the role of people with cancer in their own care. To enhance cancer control, a focus on prevention and early detection is important; however, more mAPP interventions related to cancer treatment, follow-up and survivorship are also needed to enable more cost-effective cancer care.

## Introduction

Cancer is a public health problem, contributing significantly to morbidity, mortality, disability and economic burden in low- and middle-income countries (LMICs) [1]. In 2020, it was estimated that 70% of 10 million cancer deaths were recorded in LMICs [2]. It is projected that three-quarters of all cancer deaths will occur in LMICs by 2030 [3]. The American Cancer Society defines cancer control as any evidence-based intervention that focuses on reducing the incidence, morbidity, mortality of cancer and/or improve the quality of life for people with cancer [4]. Screening, prevention, early detection, diagnosis, treatment and palliative care interventions are important to enhance cancer control. A recent study identified five priority areas for cancer research in LMICs, namely: reducing the burden of patients diagnosed with advanced-stage cancers; improving the access to, affordability of and outcomes of cancer treatment; value-based care and health economics; quality improvement and implementation research; and leveraging technology to improve cancer control [5]. It is imperative to provide information and support people living with cancer, their families and friends to guide decision making [6].

The World Health Organisation (WHO) defines mobile health as medical and public health practices supported by mobile devices, such as mobile phones, personal digital assistants, patient monitoring devices and other wireless devices [7]. The WHO has indicated that mobile-based applications (mAPP) are not a substitute to health workforce, access to essential medicines, financing, leadership and governance, which are the fundamental components of health systems [8]. However, in cancer control, mAPP interventions can be designed to: promote cancer prevention messages; facilitate access to screening services; allow for quick notification of medical imaging and/or laboratory results to facilitate diagnostic decision-making; support treatment adherence; and promote follow-up, survivorship and palliative care [9–12]. Key principles of mobile technology to optimise cancer control are related to health communication, self-management and social support [13].

Many studies from high-income countries (HICs) have demonstrated the association of using mAPP with better symptom management, enhanced communication between patients and providers, increased compliance to reporting treatment-related toxicity, improved quality of life and increased physical activity [14–20]. Majority of households in LMICs have access to at least one mobile phone [21]. The explosion of mobile phone usage in LMICs has the potential to reduce barriers to services for hard-to-reach populations by: promoting cancer awareness messages; providing real-time cancer prevention information; and providing online diagnostic and treatment support; and providing support to individuals living with and beyond cancer to self-manage the emotional, physical and socioeconomic effects of the disease and it care [22, 23].

Several rapid and systematic reviews, mostly focused on HICs, have examined mAPP interventions for: breast cancer patients/survivorship [24, 25]; cancer screening [26]; chemotherapy-related symptoms and management [27]; and chronic disease monitoring [28]. Accordingly, the aim of this systematic review was to identify and appraise mAPP interventions that have been used to support cancer control and care in LMICs.

## Methods

This systematic review was conducted and reported in accordance with the Preferred Reporting Items for Systematic Reviews and Meta-Analyses (PRISMA) guidelines [29].

### Data Source and Search Strategy

A comprehensive search was conducted for relevant studies in the following electronic databases: Cochrane Library; CINAHL; EMBASE and MEDLINE. These electronic databases were selected because they are continuously updated with new publications. Databases were searched between 28th July 2022 and 8th September 2022. The search strategy included terms relating to the following concepts: cancer; mAPP; and LMICs. Medical subject headings, keywords and free text terms were combined using “AND” or “OR” Boolean operators. Hand search through Google and tracing of references of all articles included were searched for additional studies. The search strategy was first developed for MEDLINE (Ovid) and modified in other databases when needed (see Supplementary Material S1).

### Inclusion and Exclusion Criteria

The inclusion criteria limited admission to those studies that: reported primary research findings about mAPP interventions to control cancer; conducted in LMICs; and were published in the English language, with no date limit. Editorials, commentaries, non-original articles, studies without full texts, studies not focusing on cancer control and care, studies conducted in HICs and articles published in languages other than English were excluded. The World Bank Group categorises LMICs into low-income countries (those with a gross national income (GNI) *per capita* of $1,085 or less), low-middle income countries (those with a GNI *per capita* between $1,086 and $4,255), and upper-middle-income countries (those with a GNI *per capita* between $4,256 and $13,205). A total of 136 countries are combined to represent LMICs, with 28 low-middle countries, 54 low-middle income countries and 54 upper-middle-income countries [30].

### Study Selection

**FIGURE 1:**
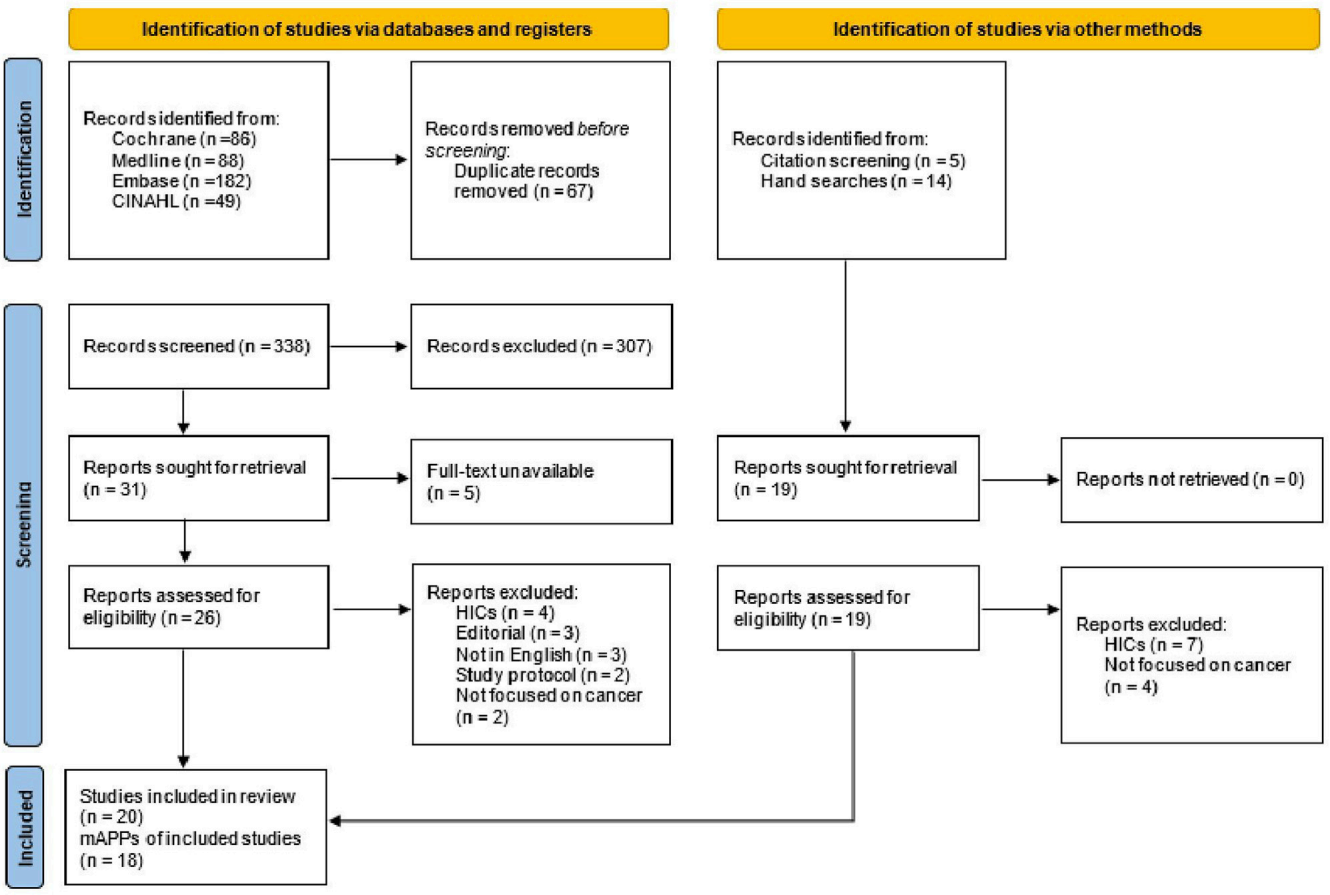
Preferred reporting items for systematic reviews and meta-analyses flow diagram (low- and middle-income countries, 2014–2022).

Following the search of the electronic databases, all citations of the identified records were collated and uploaded into the EndNote Version 20 reference manager for removal of duplicated files and storage. The titles and abstracts of the articles were screened by two independent review authors (JA and DK-M) for relevance. The two reviewers then reconciled the outcome of the screening. Full-text of potential articles assessed as relevant on the abstract review were retrieved and screened by the same independent reviewer authors against the inclusion criteria. Full-text articles that did not meet the inclusion criteria were excluded and reasons for exclusion were justified. Any disagreements that occurred between the two review authors were resolved through mutual discussion and where no consensus was reached, a third reviewer (AD) was involved. The review articles selection process is further detailed in the PRISMA flow diagram (Figure 1).

### Data Extraction

Three review authors (EO, JA, and AD) independently extracted data such as study characteristics (first authors, publication year, country, study aim and design, age group, participants, cancer type and sample size), key study findings, mAPP name, purpose of the mAPP, main features of the mAPP, privacy and security, platform and easy-to-use. According to the mAPP intervention’s purpose, studies were categorised as providing screening, prevention, early detection, diagnosis, treatment, follow-up, survivorship, supportive and palliative care.

### Study Quality Assessment

Two review authors (DK-M and AD) and a further researcher (SO) assessed the quality of the included studies. Quantitative studies were assessed according to the appropriate Joanna Briggs Institute Critical Appraisal Checklist, such as cross-sectional studies and cohort studies [31, 32]. Qualitative studies were assessed by using the Joanna Briggs Institute Critical Appraisal Checklist for Qualitative Research [32]. Discrepancies were resolved by discussion. Adapted

### Data Synthesis

Due to the range of study designs and outcomes involved, a narrative synthesis using approaches described by Popay et al. was performed without meta-analysis [33]. The Mhealth Index and Navigation Database (MIND) framework has the following criteria: Accessibility; privacy and security; clinical foundation; engagement style; and therapeutic goal [34]. Although MIND focuses on mental health mAPP interventions, its considerations and categories are transferable to health mAPP interventions more broadly [35]. The modified MIND framework was approved as the analytical framework for this review because it offers a useful tool to appraise cancer mAPP interventions, with criteria such as: privacy and security; input and outputs; evidence base and clinical foundation; interoperability and data sharing; mAPP origin and functionality; features and engagement; and ease of use [35]. Privacy and security relate safety systems available and a description of how the mAPP uses protected health information. Interoperability and data sharing relate to the mAPP ability to connect and communicate with different systems and devices in a coordinated way, without effort from the user. Evidence base and clinical foundation evaluate if the mAPP has been researched and if there is a theory driving the mAPP development. The origin and functionality relate to the mAPP availability across platforms, cost and offline functionality. Ease of use indicates how easily users can use a the mAPP [35]. The modified MIND framework criteria are generic and applicable to cancer mAPP interventions. Information from the included studies was independently coded by two reviewers (AD and EO) and mapped against the modified MIND framework. The extracted information from the studies was read and reread to identify common themes. Any discrepancies were resolved through discussion.

## Results

An initial search through the electronic databases yielded 405 studies, of which 67 duplicates were removed. The 338 remaining articles were screened by title and abstract and 307 articles were excluded using the exclusion criteria. Of the remaining 31 articles, 12 articles met the inclusion criteria. An additional 8 articles were included through reference tracing and hand searches. Finally, a total of 20 articles reporting on 18 cancer mAPP interventions were included in this review. Two articles reported on the ColorAPP [36, 37] and another two on e-Support intervention [38, 39] (see Figure 1).

### Characteristics of the Included Studies

**FIGURE 2:**
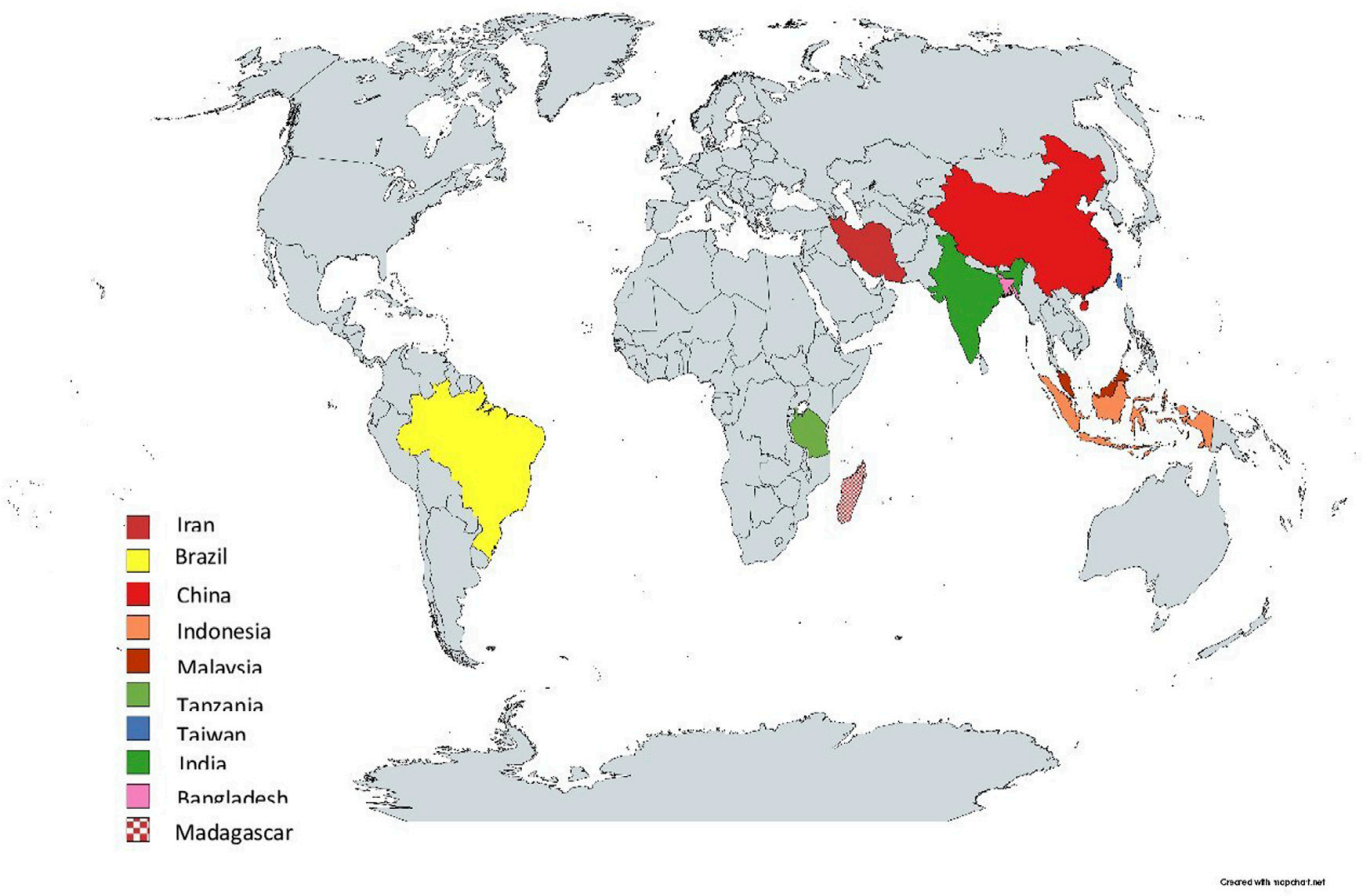
Geographical distribution of the included studies across three countries (low- and middle-income countries, 2014–2022).

Out of the 20 articles included in this review published between 2014 and 2022, 13 used quantitative [37, 38, 40–50], six used mixed-methods study designs [36, 51–55] and one used qualitative [39]. Participants included cancer patients, family members, community members and healthcare professionals, with a cumulative sample size of 33,615 ranging from 13 [39] to 22,337 [50]. The mean age was 52.57 ± 9.37. The studies were conducted across 10 countries, namely: China [38, 39, 41, 45, 47]; Iran [40, 42, 51, 53]; Brazil [43, 48, 54]; Malaysia [36, 37]; Bangladesh [50]; India [52]; Indonesia [44]; Tanzania [46]; Taiwan [55]; and Madagascar [49] (see Table 1). Figure 2 shows the geographical distribution of the identified studies.

**TABLE 1:** Characteristics of included studies (low- and middle-income countries, 2014–2022).

Authors/Year	Country	Study aim	Study design	Sampling type	Sample size/Follow-up/Attrition rate	Gender	Mean age	Other sociodemographic characteristics	Key findings
Salmani, Nahvijou [41]	Iran	To develop and evaluate the usability of a smartphone-based application for the self-management of patients with colorectal cancer	Cross-sectional	Convenience	17 colorectal cancer patients	10 females 7 males	57.18 ± 17.47	Education:	• Good usability level of the mAPP
								• Diploma and undergraduate education = 13	• Mean usability to screen design and layout was 8.18 out of 10
								• Associate degree = 1	• Mean usability to the terminology and systems information was 7.97 out of 10
								• Bachelor = 2 • Master’s degree = 1	• Mean learnability was 7.98 out of 10
									• Mean usability to the system features was 8.12 out of 10
Rezaee, Asadi [54]	Iran	To develop and evaluate the usability and quality of an educational mHealth app aimed at improving the resilience of breast cancer in women	Mixed methods	Convenience	25 women with breast cancer	All females (patients)	30–60 years	Education: • No university education = 15	• High satisfaction with the usefulness and the probability of recommending it to other cancer survivors (mean score of 83.20)
					Four expert participants (medical informatics specialist, hematology and oncology specialist, medical education specialist and cognitive neuroscience specialist)			• University education = 10	• Mean learnability score was 84.80 out of 100 (94.14–75.45), with a standard deviation of 7.52
									• Mean usability score was 81.60 out of 100 (86.04–77.15), with standard deviation of 3.57
									• Subjective quality mean score was 3.42
Cavalcante Pires, Cezar [55]	Brazil	To implement a mobile application for cancer care management at the Brazilian National Cancer Institute	Mixed methods	Convenience	50 participants (patients and family members)	Not reported	Not reported	Not reported	• Cancer patient information readily made available to health professionals
									• Cancer patients receive instant notifications of appointment, medication prescription and educational messages
Ayyoubzadeh, Shirkhoda [52]	Iran	To identify and analyse the required features of remote monitoring smartphone apps designed to follow up colorectal cancer survivors with the focus of supporting them after surgery	Cross-sectional, mixed methods	Random	27 participants (18 health professional and 9 colorectal cancer survivors)	8 females 19 males	50–79 years	Specialty: • General surgeon = 5 • Oncology surgeon fellowship = 7 • Clinical oncologist = 3 • Other = 3 Work experience • Less than 5 years = 3 • Between 5 and 10 years = 11 • Between 11 and 15 years = 1 • Between 16 and 20 years = 1 • More than 20 years = 2	• Lack of availability of smartphone
									• The need for more information on colostomy bags
									• The need for a feature to send laboratory results to clinician via the mAPP
Wang, Ye [42]	China	To explore the effects of a “Shared Decision-Making Assistant” smartphone application on the decision-making of informed patients with primary liver cancer in China	Quasi-experimental	Simple random	180 participants (90 patients in the intervention group; and 90 patients in the control group) Follow-up: 3 months Attrition rate: 5.3%	Intervention (22 females; 68 males) Control (19 females; 71 males)	50.0 ± 9.03 intervention group 51.7 ± 8.39 control group	Marital status:	Mean effect size for outcome variables (control vs. intervention): • Decision conflict: 26.75 ± 9.79 vs. 16.89 ± 8.80, *p* < 0.001 • Uncertainty: 5.61 ± 2.69 vs. 2.73 ± 2.44, *p* < 0.001 • Clarity: 7.48 ± 3.37 vs. 4.25 ± 3.40, *p* < 0.001 • Decision preparation: 63.84 ± 7.38 vs. 80.73 ± 8.16, *p* < 0.001 • Decision self-efficacy: (76.89 ± 13.46 vs. 87.75 ± 6.87, *p* < 0.001 • Knowledge: (12.72 ± 2.13 vs. 14.52 ± 1.91, *p* < 0.001
								• Intervention group (69 married; 21 others)	
								• Control group (64 married; 26 others)	
								Residential location:	
								• Intervention group (61 city; 29 village)	
								• Control group (51 city; 39 village)	
								Religious affiliation	
								• Intervention group (11 yes; 79 no)	
								• Control group (16 yes; 74 no)	
								Education:	
								• Intervention (10 primary; 26 junior high; 32 senior high; 22 college or above)	
								• Control (16 primary; 33 junior high; 28 senior high; 13 college or above)	
Shakery, Mehrabi [43]	Iran	To determine the effect of a smartphone application on women’s performance and health beliefs regarding BSE	Quasi-experimental	Simple random	140 participants (65 patients in the intervention group; and 75 patients in the control group) Follow-up: 4 months Attrition rate: 13.3%	All females	36.9 ± 10.5	Education • Intervention group (8 below diploma; 15 diploma; 9 associate degree; 33 bachelor and higher degrees) • Control group (17 below diploma; 16 diploma; 9 associate degree; 33 bachelor and higher degrees) Marital status • Intervention group (48 married; 17 single) • Control group (65 married; 10 single)	Mean effect size for outcome variables (control vs. intervention):
									• Perceived susceptibility: 12.09 ± 2.68 vs. 12.49 ± 2.19, *p* = 0.445
									• Perceived severity: 19.81 ± 5.75 vs. 21.35 ± 5.35, *p* = 0.105
									• Breast self-examination benefits: 23.77 ± 3.37 vs. 24.06 ± 3.90, *p* = 0.640
									• Breast self-examination barriers: 33.94 ± 4.84 vs. 37.33 ± 4.76, *p* < 0.001
									• Self-efficacy: 23.52 ± 6.46 vs. 36.31 ± 7.62, *p* < 0.001
									• Health motivation: 25.32 ± 4.49 vs. 28.51 ± 3.58, *p* < 0.001
Cavalcanti, Bushatsky [44]	Brazil	To evaluate the usability of a mobile application for early detection of pediatric cancer	Descriptive quantitative	Convenience	19 nurses	All females	24–69 years	Not reported	• Mean score of easy-to-use system when used for the first time (90.46 out of 100)
									• Mean score of speed in the execution of the established tasks (91.2 out of 100)
									• Absence or low error rate (90.79 out of 100)
									• Mean score of easy to execute system even after a long period without using it (96.05 out of 100)
									• Mean score of pleasant design (88.6 out of 100)
Adiyasa and Wirata [45]	Indonesia	To determine the effect of a BSE application android called BECA on BSE Practice	Quasi-experimental	Purposive	32 community women Follow-up: 1 month Attrition rate: 0%	All females	20–50 years	Marital status	Frequency of breast self-examination practice (pre-test vs. post-test: • Not practice: 32 vs. 8 • Practice: 0 vs. 24
								• Single = 6	
								• Married = 22	
								• Widow = 4	
								Education	
								• Junior high = 3	
								• Senior high = 18	
								• Diploma = 5	
								• Bachelor = 5	
								• Magister = 1	
Zhu, Chen [39]	China	To examine the usage duration and login frequency of an app-based intervention, the Breast Cancer e-Support (BCS) program and to investigate the association between usage data and participants’ demographic and medical characteristics	Randomized controlled trial (secondary data analysis)	Random	57 breast cancer patients	All females	46.2 ± 8.5	Not reported	• The Discussion Forum and the Learning Forum were the most popular forums for women to log in and use • Age, education, family monthly income and employment were associated with BCS usage duration and/or login frequency (*p* > 0.5)
Yaacob, Mohamad Marzuki [38]	Malaysia	To assess the effectiveness of the ColorApp mobile app in improving the knowledge and attitude on colorectal cancer among users aged 50 years and older, who are the population at risk for the disease in Kedah	Quasi-experimental	Simple random	100 community members (50 in the intervention group; and 50 in the control group) Follow-up: 2 weeks Attrition rate: 0%	50 females 50 males	56.0 ± 5.6 intervention group 55.8 ± 4.76 control group	Education • Intervention group (7 tertiary; 33 secondary; 10 primary) • Control group (3 tertiary; 35 secondary; 12 primary) Current occupation • Intervention group (17 employed; 33 unemployed) • Control group (22 employed; 28 unemployed)	Adjusted mean effect size for outcome variables (control vs. intervention):
									• Knowledge score: 0.66 95% CI (−0.91, 2.23), *p* = 0.40) vs. 2.65 95% CI (0.75, 4.55), *p* = 0.007
									• Attitude score: −3.00 95% CI (−7.65, 1.65), *p* = 0.20 vs. 0.77 95% CI (−2.17, 3.70), *p* = 0.60
Wang, Chen [46]	China	To develop an application dynamically monitoring the prostate cancer (Pca) risk for patients to assess their own progression of Pca risk at home	Cohort	Purposive	1,553 prostate cancer patients	All males	68.94 ± 8.15	Not reported	• Application showed decent performance in predicting the risk of Pca and clinicopathology (>95%) • Convenient for patients to self-assess the progress of Pca risks
Rubagumya, Nyagabona [47]	Tanzania	To explore the feasibility of using a mobile app for detection of skin cancers in people with albinism in Tanzania	Prospective	Convenience	69 people with albinism presenting with skin lesions	26 females 43 males	47 6 ± 10.98	Not reported	• 77 lesions from different body locations were captured by the NgoziYangu mAPP • 62 lesions (81%) were considered malignant via the mAPP
Hou, Lan [56]	Taiwan	To investigate the information needs of Taiwanese women with breast cancer to inform the development of a self-management support mHealth app	A 5-step design thinking approach	Purposive	15 breast cancer patients	All females	55.3 ± 8.5	Education	• 8 major themes were identified, namely: treatment, physical activity; emotion; diet; health records; social resources; experience sharing; and expert consulting
								• Junior high = 5	
								• Senior high = 4	
								• College = 5	
								• Graduate = 1	
								Marital status	
								• single = 1	
								• Married = 11	
								• Widow = 3	
Cheng, Ho [48]	China	To evaluate the feasibility, safety, and efficacy of a comprehensive intervention model using a mobile health system (CIMmH) in patients with esophageal cancer after esophagectomy	Prospective, single arm pilot	Purposive	20 esophageal cancer patients Follow-up: 3 months Attrition rate: 20%	2 females 18 males	62.2 ± 7.1	Residential location	mean effect size for outcome variables (baseline vs. 3 months follow-up): • Overall quality of life: 76.70 ± 17.40 vs. 69.80 ± 12.10
								• City = 8	
								• Rural = 12	
								Education	
								• Less than high school = 11	
								• High school or greater = 9	
								Marital status	
								• Married = 20	
Marzuki, Yaacob [37]	Malaysia	To document the process of designing and developing a mobile app for community education on colorectal cancer and assess the usability of the prototype	Mixed methods (focused group and cross-sectional)	Purposive (qualitative) Simple random (quantitative)	11 expert participants (public health physician, gastroenterologist, one family medicine physician, medical officers, assistant environmental health officer, individuals from intended users and researchers) 50 community members never diagnosed with any cancer	25 females (for the 50 participants)	56.0 ± 5.69 (for the 50 participants)	Education • Primary = 10 • Secondary = 33 • Tertiary = 7 Occupation • Unemployed = 2 • Self-employed = 12 • Retired = 1 • Clerical work = 32 • Professional = 3	Focus group participants agreed on: the following topics:
									• Introduction to colorectal cancer
									• Sign and symptoms
									• Risk factors
									• Prevention
									• Colorectal cancer screening program
									Usability score for ColorApp prototype showed a mean difference of 4.9 (*p* = 0.004; 95% CI 1.626–8.174)
Goulart Silveira, Carcano [49]	Brazil	To determine whether the diagnosis of suspected skin cancer lesions performed using digital photography with a conventional smartphone application that has been designed exclusively for this purpose was accurate and reliable in comparison to the findings of the face-to-face consultations	Prospective		39 individuals monitored by routine skin cancer screening	27 females 12 males	68	Not reported	• Lesions were mostly found on the face (69%), followed by upper limbs (15%), scalp (8%), trunk (6%) and lower limbs (2%) • 71% of lesions were malignant, with 32% being squamous cell carcinoma (SCC) and 68% being classified as basal cell carcinoma (BCC) and 29% were considered benign
Zhu, Ebert [40]	China	To explore the participants’ perception of breast cancer e-Support program, its strengths and weaknesses and suggestions to improve the program	Descriptive qualitative	Purposive	13 breast cancer patients	All females	49.5 ± 9.5	Marital status:	• Four main themes were identified, namely: benefits of breast cancer e-Support; challenges to engagement; suggested improvement; and future direction
								• Married = 13	
								Education:	
								• University level or above = 4	
								• High school = 6	
								• Middle school = 2	
								• Primary school = 1	
								Employment status	
								• Employed = 3	
								• Unemployed = 10	
Quercia, Tran [50]	Madagascar	To assess the feasibility of a mobile health data collection system to facilitate monitoring of women participating in a cervical cancer screening campaign	Cross-sectional	Convenience	151 individuals attending Saint Damien Health Centre	All females	41.8 ± 9.1	Marital status	• 12% had knowledge about cervical cancer and none of them reported a family history of cervical cancer • 1.3% knew about their HIV status
								• Single = 35	
								• Married = 116	
								Employment status	
								• Housewife = 18	
								• Sales assistant = 19	
								• Farmer = 90	
								• Others = 23	
								Education:	
								• None = 35	
								• Elementary school = 68	
								• High school = 46	
								• University = 2	
Bhatt, Isaac [53]	India	To determine: the key features of an ideal mHealth prototype for use in cancer screening in LMIC settings; the views of community health workers, nurses and others involved in the delivery of the programme, on feasibility of using the prototype, how acceptable they found it, and how it might be improved; and the response of the target population to screening invitations from the programme	Mixed methods	Convenience	8,686 women screened for cervical and oral cancers	All females	Not reported	Not reported	• Of the 170 women who were screened for cervical cancer, 49 (28%) tested positive on VIA • Among those (8,516) who were screened for oral cancer, 5% (*n* = 490) tested positive, but only 151 (30.8%) attended for follow-up
					2 focus group discussions with 12 participants				
					Interviews with 8 informants				
Ginsburg, Chowdhury [51]	Bangladesh	To demonstrate proof of concept for a smart phone empowered community health worker model of care for breast health promotion, clinical breast examination and patient navigation in rural Bangladesh	Three arm randomized controlled trial	Random	22,337 participants (7,827 Arm A; 7,526 Arm B; and 6,984 Arm C) Follow-up: 1 month Attrition rate: 1%	All females	38.0 ± 11.2 overall (37.4 ± 10.7 Arm A; 38.3 ± 10.6 Arm B; 38.4 ± 12.4 Arm C)	Education:	• A total of 556 of 22,337 women had an abnormal CBE, of which only 70 were from the control arm • Women in arm B (smartphones plus patient navigation) were significantly more likely to attend for care versus women in arm A (smart phones without navigation; 63% vs. 43%, *p* <0.0001)
								• Arm A (2,000 no education; 4,874 primary; 862 secondary; 91 missing data)	
								• Arm B (1,595 no education; 5,190 primary; 683 secondary; 58 missing data)	
								• Arm C (929 no education; 4,154 primary; 542 secondary; 1,359 missing data)	

### mAPP Interventions Used Across the Cancer Control and Care Continuum

Supplementary Table S1 shows the characteristics of identified cancer control and care mAPP interventions. Majority of the studies (*n* = 15) did not have a theoretical framework guiding the mAPP intervention design, implementation and/or evaluation. Identified mAPP interventions focused on breast cancer (*n* = 6) [38, 42, 44, 50, 53, 55], colorectal cancer (*n* = 3) [37, 40, 51], cervical cancer (*n* = 2) [49, 52], skin cancer (*n* = 2) [46, 48], oesophageal cancer (*n* = 1) [47], liver cancer (*n* = 1) [41], prostate cancer (*n* = 1) [45], paediatric cancers (*n* = 1) [43] and general cancers (*n* = 1) [54]. More than half (*n* = 10) of the identified 18 mAPP interventions aimed at optimizing prevention, screening and early detection of cancer [36, 37, 42–46, 48–50, 52]. Almost one-third (*n* = 5) of the mAPP interventions focused on improving supportive and palliative care for adults with cancer [38, 40, 47, 53, 55] and two mAPP interventions were designed to help people with cancer make informed treatment decisions and maintain autonomy [41, 54]. Only one mAPP intervention was developed and implemented to facilitate follow-up and survivorship care for people with colorectal cancer in Iran [51].

### Mobile Platforms for Engaging Users

Nine of the mAPP interventions were compatible with only Android [37, 40–44, 50, 53, 55] and two were compatible with both Android and iPhone operating systems (iOS) [38, 54]. Four of the mAPP interventions combined the features of both web-based and Android/iOS [46, 48–50]. However, four studies did not report whether their mAPP interventions were built on a single or cross-platform to engage users [45, 47, 51, 52].

### mAPP to Improve Breast Cancer Prevention and Early Detection Knowledge

Two breast cancer prevention and early detection mAPP interventions included information about breast cancer (e.g., risk factors, awareness of early signs and symptoms and prevention strategies), breast self-examination training videos and a monthly breast self-exam reminder system [42, 44]. One quasi-experimental study assessed the effect of an mAPP (*n* = 65) compared to no intervention (*n* = 75) on participants’ performance and health beliefs about breast self-examination. The mean differences of the scores of perceived susceptibility (1.03 ± 2.65 vs. 0.01 ± 0.42, *p* = 0.001), breast self-examination barriers (2.80 ± 5.32 vs. 0.04 ± 1.43, *p* = 0.001), self-efficacy (10.75 ± 7.63 vs. −2.75 ± 2.44, *p* = 0.001) and health motivation (2.77 ± 3.70 vs. −0.29 ± 0.63, *p* = 0.001) were significantly higher in the mAPP group compared to the control group [42].

### mAPP for Early Detection of Skin Cancer

The most common features of two skin cancer screening and early detection mAPP interventions were: camera for digital imaging of skin lesions; clinical history and characterization of skin lesions; and web portal for a dermatologist to provide diagnostic opinion [46, 48]. One feasibility study reported that 81% of 77 lesions from 69 participants captured by an mAPP were considered malignant and referred for biopsy and histologic diagnosis [46]. The histopathological findings showed that 85% of the 55 lesions biopsied were confirmed as skin malignancies [46].

### mAPP to Navigate Cancer Treatment Decisions

Key features of mAPP interventions to support people with cancer navigate treatment decision-making included: epidemiological information; laboratory examination information; treatment-related information; and patient-clinician interactive platform [41, 54]. Findings from a quasi-experimental study from China revealed that participants in an mAPP intervention group named “Shared Decision-Making Assistant” had significantly higher decision preparation score (80.73 ± 8.16 vs. 63.84 ± 7.38; *p* = 0.001), decision self-efficacy score (87.75 ± 6.87 vs. 76.89 ± 13.46; *p* = 0.001), decision satisfaction score (25.68 ± 2.10 vs. 23.12 ± 3.91; *p* = 0.001) and knowledge of primary liver cancer treatment score (14.52 ± 1.91 vs. 12.72 ± 2.13; *p* = 0.001) than those of the control group [41]. The study also reported significant lower decision conflict scores for participants in the intervention group compared to those in the control group (16.89 ± 8.80 vs. control group: 26.75 ± 9.79; *p* < 0.05) after 3 months [41].

### mAPP for Supportive and Palliative Care

Supportive and palliative care mAPP interventions had features such as: psychological support [40, 47, 55]; nutrition/diet guidelines [40, 47, 55]; physical activity [40, 47]; patient educational content [38, 40]; online community for people with cancer and their families for social support [38, 47]; self-care platform for monitoring physical symptoms (e.g., pain) and sharing experiences [38, 40, 55]; and medication chart to enable safe and accountable medication management [40]. It was identified that psychological techniques including mental support, music therapy, meditation, good sleep and relaxation exercises help to ease anxiety, distress or sadness the person with cancer may feel [40, 47, 55].

### Easy-to-Use mAPP Intervention

Easy-to-use mAPP was the most commonly identified usability dimension, which was assessed in eight studies [36, 40, 42–44, 51–53]. The review identified that highly educated participants perceived mAPP interventions as easy and effortless to use. For example, a study among Brazilian nurses to evaluate the usability of an mAPP for early detection of paediatric cancer reported:

“Specialist nurses considered the software as an easy-to-use, accessible, practical device, with very well-grounded content and very useful in the assistance-teaching-learning process” [43].

Studies reported benefits that older adults with cancer derived from mAPP interventions. These benefits included: medication reminders [40]; high-quality remote consultation [51]; and timely provision of psychosocial support [40, 55]. However, the review recognized that older adults with cancer face challenges in learning and utilizing mAPP interventions. Family members needed to support and guide the elderly cancer survivors to learn and use mAPP interventions. For example, an Iranian participant narrated:

“An older adult survivor could not work with the app… a member of the family may help and work with the system instead” [51].

Other identified dimensions of cancer mAPP usability included: layout and interface quality [36, 40, 43, 53]; learnability [36, 40, 53]; system quality [36, 43, 53]; simplicity and understandable [36, 43, 53]; user-friendly [36, 53]; tailored communication [36, 53]; coordination [36, 53]; technical support [36, 53]; terminology [40]; and task execution [43].

### Privacy and Security Measures of the mAPP Intervention

All the identified mAPP interventions collected and tracked users’ data to help improve early detection, enhance cancer treatment and optimize palliative care. More than half (*n* = 10) of the included studies stored the data on users’ devices [37, 38, 40–44, 50, 53–55]. Four studies stored them on both the server and users’ devices [38, 50, 53, 54] and one study stored all the data on the server [49]. The review identified that storing users’ information behind password-protected encrypted channels was imperative to ensure privacy and security. For example, one study that used mAPP intervention to monitor women participating in cervical cancer screening campaign in Madagascar reported:

“For security and privacy, data transfer to central database was done using an encryption method… Authentication was required to access the patients’ files, and only caregivers who had received a personal identifier and password could log in the smartphone application or the Medical Unit. Access to patient data was made possible by scanning a unique bar code for each patient or by entering the patient’s full name, thus ensuring patients’ data protection” [49].

### Instruments Used to Assess mAPP Interventions

Sixteen validated assessment tools were identified in the included studies. One study used the Questionnaire for User Interaction Satisfaction version 5.5 to evaluate the usability of a smartphone-based application for the self-management of people with colorectal cancer [40]. The System Usability Scale was employed to evaluate the usability of an mAPP developed for early detection of paediatric cancer in Brazil [43], the quality of an educational mAPP aimed at improving the resilience of people with breast cancer in Iran [53] and an mAPP focused on community education on colorectal cancer [36]. Tools that were adopted to evaluate the feasibility, safety and efficacy of mAPP intervention to improve the quality of life of people with oesophageal cancer included: European Organization for Research and Treatment of Cancer-Quality of life Question-Core; European Organization for Research and Treatment of Cancer-Quality of life Question-Oesophageal Cancer Module; Chinese versions of Patient Health Questionnaire-9; General Anxiety Disorder-7; and Perceived Stress Scale-10 [47]. The study that developed the mAPP intervention to assist the decision-making needs of people with primary liver cancer used six tools: Decisional Conflict Scale; Preparation Decision Making Scale; O’Connor’s 11-item Decision Self-Efficacy Scale; Satisfaction with Decision Scale; Breast Cancer Knowledge Scale; and Decision Regret Scale [41]. The Champion’s Health Belief Model Scale was used by one study to determine the effect of an mAPP intervention on women’s performance and health beliefs regarding breast self-examination [42].

### Quality Assessment

Six of the quantitative studies adopted instruments with acceptable validity and reliability to measure outcomes [37, 40–43, 47]. All the studies described the settings and eligible participants. Two of the included studies did not report whether or not they secured ethics approval [51, 54]. Five of the included studies explicitly indicated that extensive literature review was conducted to iteratively develop the cancer mAPP interventions [39–41, 51, 55]. The included qualitative studies did not have a statement locating the researchers culturally or theoretically. The outputs of the integration of qualitative and quantitative components of three of the mixed method studies were not adequately interpreted [51, 53, 54] (see Supplementary Material S2).

## Discussion

This systematic review provides evidence on mAPP interventions developed and implemented to help cancer control and care in LMICs. Currently, there is a relatively small number of studies available for analysis but growth in research in cancer control and care mAPP interventions is to be expected. Studies evaluating mAPP cancer control and care interventions effectiveness is scarce. Future rigorous studies are needed to adequately evaluate which features makes the mAPP intervention more user-friendly to promote cancer control and care. The review results showed that most studies did not have a theoretical framework guiding the design, implementation and/or evaluation of mAPP interventions. Without a theoretical framework, mAPP interventions may be perceived by the target population as ineffective in promoting health behaviour [56].

Nearly half of the included studies developed, implemented and/or evaluated cancer control and care mAPP interventions were only available for Android operating system. Since Android operating system dominates the market in China, Iran, Africa and most LMICs, this is an expected finding that shows the difference in digital landscape with many HICs. By comparison, a review of mAPP interventions to promote the management of COVID-19 in a HIC—Spain—found that: 8% mAPP were available for Android; 38% available for iOS; and 54% available for both Android and iOS [57].

Usability has been defined as the extent to which a user can utilise a product to achieve a specific goal with satisfaction, effectiveness and efficiency in a specified context [58]. Several usability dimensions have been documented in the literature. They include system quality, tailored communication, trust, regularly updated information, information quality, perceived usefulness, interface quality, terminologies, understandability and easy-to-use mAPP intervention [59, 60]. The review confirms the existing literature in finding that with higher education levels users tend to find it easy to navigate and use mAPP interventions [61, 62]. Older people with cancer often face additional challenges in using mAPP intervention; however, digital literacy will likely improve among older people as the generations move forward.

Mobile apps can be positioned throughout the cancer control and care continuum. Since the intention is for persons with an interest in or who have cancer to be able to access information, cancer control and care mAPP interventions in conjunction with other measures should always be relevant to the local environment and provide information in a logical manner. This will help avoid confusion and improve user understandability to perform the required tasks. To create an appropriate mAPP for LMICs, the recommended five steps framework can be followed: characterize the problem and the target user; review the literature; translate information to knowledge; protect information; and evaluate usability and efficacy [63]. Future studies can assess the impact of cancer mAPP interventions based on: reach (downloads and subscriptions); user engagement and experience; effectiveness (impact on health beliefs, behaviour and lifestyle to cancer prevention, early detection, diagnosis, treatment and palliative care); adoption; implementation (adaption, consistency and cost); and maintenance (long-term effectiveness and implementation).

For optimal trust and functionality of cancer control and care mAPP interventions, the issue of data privacy and security must be addressed. The main privacy and security issues are related to verification, authorization, access control, system configuration, information storage and management [64]. The review results indicate that developers of cancer control and care mAPP interventions should consider a wide range of security solutions, including encryption and identity management practices that protect data across platforms. Also, people with cancer, their family members and healthcare professionals should be involved in the development of cancer control and care mAPP interventions to enhance acceptability [65].

Features or functionalities of the cancer control and care mAPP typically depend on the purpose of the application. Studies within the current review focused on mobile technologies to prevent, screen and for early detection of cancers constituted 59% of all identified mAPP interventions. Major features of mAPP interventions related to this early phase of the cancer process included: reminders; calendar and appointment tracking; videos to facilitate awareness; and easy-to-use information [36, 37, 43]. Recent studies from HICs have indicated that a good mAPP can empower patients by providing useful information that enables them to manage cancer control and care barriers [66–68]. Supportive and palliative care mAPP interventions may play a crucial role in helping people with cancer manage symptoms and medications and communicate with their care team. Other identified features of supportive and palliative care mAPP interventions in the current review included: dietary guidelines that provide up-to-date advice about the amount and kinds of foods that people with cancer need to eat for wellbeing; links to relevant websites for educational resources; and self-care advice.

The review identified that across LMICs, few studies have aimed at developing mAPP interventions to enhance access to cancer treatment, follow-up and survivorship care. It was however revealed that cancer treatment mAPP interventions are useful for supporting shared decision-making. Cancer treatment mAPP features that can support people with cancer include: writing notes regarding treatment (surgery, radiotherapy and chemotherapy) side effects; recording conversations and answers from healthcare professionals; and documenting concerns to use for follow-up [65].

The results of this review support the WHO recommendations that digital interventions should complement and enhance health system functions through timely exchange of information where patient safety, privacy, traceability, accountability and security can be monitored [8]. The development of mAPPs is both necessary and inevitable across the globe but more particularly in LMICs where the cancer burden is growing and efforts to impact on cancer control and care are imperative. Features covered in this article apply across the domains of cancer and can be used to guide such interventions. Most LMICs have internet challenges, including: unavailability of internet; slow internet connectivity; and high internet cost which may limit widespread implementation.

### Strengths and Limitations

To the best of our knowledge, this is the first systematic review of the available literature on mAPP interventions implemented to help control and manage cancer in LMICs. The review was conducted using rigorous systematic literature search methods. The limitations are that the review did not search app stores, so useful cancer control and care mAPP interventions not published in peer-review journals may have been overlooked. At the same time, however, other reviews have found that mAPP interventions that have not been published are unlikely to have been extensively user-tested and often have limited usability [69]. Perhaps most importantly, while several of the mAPP interventions we identified had satisfactory usability, our review could not identify robust evidence for the effectiveness of mAPP interventions in achieving positive cancer control and care outcomes in LMICs. Poor access to mobile technology, low digital skills and low health and written literacy levels were missing from the MIND framework. Finally, the available literature does not provide detailed information on data management of cancer control and care mAPPs such as the costs and number of downloads, making it difficult to ascertain how widely used the interventions are.

### Conclusion

Cancer mAPP interventions are gradually gaining attention in LMICs because they provide unique resources to empower and strengthen the participation of people with an interest in cancer or to involve those with cancer in their own care. To enhance cancer control, a focus on prevention and early detection is important; however, mAPP interventions related to cancer treatment, follow-up and survivorship are also needed to enable more cost-effective cancer care. It is recommended that future cancer control and care mAPP development, implementation and evaluation should be informed by the existing knowledge available and stakeholder engagement to improve acceptability. This research team will proceed with a study to explore application functionalities, features and potential barriers for the development of an mAPP suited to enhancing the supportive care of patients receiving radiotherapy.

## Abbreviations

LMICs, low- and middle-income countries; mAPP, mobile application; HICs, high-income countries; iOS, iPhone operating system.
