# Supplementary material for: Mobile-Based Application Interventions to Enhance Cancer Control and Care in Low- and Middle-Income Countries: A Systematic Review
Source: Int J Public Health. 2023 Dec 5;68:1606413. doi: 10.3389/ijph.2023.1606413 (PMC10732306; doi:10.3389/ijph.2023.1606413)
Supplement: Supplementary file 1 [file Table1.docx]

**Title:** Mobile-based application interventions to enhance cancer control and care in low- and middle-income countries: a systematic review

Supplementary Table 1: Characteristics of identified mobile application interventions (low- and middle-income countries, 2014 – 2022)

| **Authors and country** | **Name of mobile application** | **Cancer control and continuum** | **Cancer Type** | **Main objective of the mobile application** | **Features/Content of the mobile application** | **Platform** | **Theoretical framework** | **Implementation team institutions** | **Implementation cost** | **Geographical coverage** |
| --- | --- | --- | --- | --- | --- | --- | --- | --- | --- | --- |
| Salmani, Nahvijou (41)/Iran | Colorectal Cancer Along (ColorectAlong) | Supportive and palliative care | Colorectal cancer | To provide medication management, nutrition and diet management, mental health, smoking cessation and alcohol reduction, pain management and educational content | - Patient profile - Medication management - Nutrition and diet management - Mental health - Smoking cessation and alcohol reduction - Pain management - Educational content - Reminders, questions and notes | Android devices | Not reported | - Iran University of Medical Sciences, Tehran, Iran | Not reported | Patients from the Cancer Institute of Iran |
| Rezaee, Asadi (54)/Iran | CaRA | Supportive and palliative care | Breast cancer | To provide appropriate educational content that can improve resilience and quality of life in breast cancer patients | - Registration module - Exercise module - Question module - Evaluation module - Notification setting module - Category module - Resilience educational content:   - Understanding and awareness   - Life control   - Emotional support   - Optimism to future   - Purposefulness of life   - Importance for self and life   - Self‐confidence | Android devices | Not reported | - Shiraz University of Medical Sciences, Shiraz, Iran | Not reported | Patients from Motahari  hospital and Amir Oncology hospital in Shiraz city of Fars province |
| Cavalcante Pires, Cezar (55)/Brazil | INCA App | Treatment knowledge | General cancer | To enhance the patient-clinician relationship and guarantees patients' data consistency and accuracy | - Patient’s registration - Appointment and exams scheduling - Medical information (cancer type, diagnosis date, staging and patient's situation) - List of medication in use during treatment | Android and iOS devices | Not reported | - Instituto Nacional de Câncer, Brazil - Universidade Estácio de Sá, Brazil - Universidade do Estado do Rio de Janeiro, Brazil | Not reported | Patients from the Brazilian  National Cancer Institute |
| Ayyoubzadeh, Shirkhoda (52)/Iran | Remote monitoring smartphone app | Follow-up and survivorship care | Colorectal cancer | To help with the follow-up of patients with colorectal cancer, remote monitoring of physical and mental signs and symptoms and improving health care through patient understanding of what they need to do in each phase via an easy electronic consult with their clinical experts | - Patient information registration - Sign and symptom monitoring - Education - Physical activity - Reminders (hospital visit reminders, medication reminders) - Personalised advice (online consultation system, tailored patient information) - Patient evaluation (quality of life evaluation, nutrition evaluation and clinician-patient relationship evaluation) - Social network (patient’s discussion groups) | Not reported | Not reported | - Iran University of Medical Sciences, Tehran, Iran - Shiraz University of Medical Sciences, Shiraz, Iran - Technical University Braunschweig and Hannover Medical School, Braunschweig, Germany | Not reported | Cancer survivors who had already visited the Cancer Institute  of Imam Khomeini hospital in Tehran |
| Wang, Ye (42)/China | Shared Decision-Making Assistant application | Treatment knowledge | Liver cancer | To support decision-making of informed patients with primary liver cancer | - Primary liver cancer treatment knowledge center - Decision aids path:   - face-to-face conversation with the doctor to determine the treatment options available   - compare alternative treatment options on app that include principle, therapy pathway, indications, advantages, disadvantages, operation time, postoperative average length of hospitalization, complications, cost and 5-year recurrence rate   - explore the preferences: Patient could score the risks and benefits of different treatment options by using the Likert 5-point scoring system through the app   - knowledge testing   - another face-to-face conversation between doctors and patients to finalize the treatment plan | Android devices | Not reported | - Eastern Hepatobiliary Surgery Hospital, Naval Medical University, People’s Republic of China. - General Hospital of Northern Theater Command, Shenyang, People’s Republic of China | Not reported | Patients referred to the Eastern Hepatobiliary Surgery Hospital, Naval  Medical University, Shanghai, China |
| Shakery, Mehrabi (43)/Iran | Smartphone application | Prevention and early detection | Breast cancer | To educate women about BSE and all other related information | - Alarm system - Reminder (in form of a text message) to perform BSE based on their menstrual cycles - Video clip training BSE accurately - Four videos about breast cancer (risk factors, breast pain and discharge and prevention) - Feedback to the therapist | Android devices | Health belief model | - Shiraz University of Medical Sciences, Shiraz, Iran | Not reported | Women referred to Honari and Peymaniyeh clinics in Jahrom |
| Cavalcanti, Bushatsky (44)/Brazil | Fique Atento, pode ser cancer application | Screening and early detection | Pediatric cancer | To support early detection of pediatric cancer | - Concept of childhood cancer and its classifications - General signs and symptoms - Procedures and exams to be performed for each specificity - Diagnostics - Types of treatments - Necessary care regarding toxicity - Video lesson resources, folders, self-explanatory illustrations - Contact option for questions, compliments and suggestions | Android devices | Not reported | - Universidade de Pernambuco (UPE), Faculdade de Enfermagem Nossa Senhora das Graças. Recife, Pernambuco, Brazil - Universidade Federal de Pernambuco (UFPE), Brazil | Not reported | Nurses at the Hospital  University Oswaldo Cruz, Recife, Pernambuco |
| Adiyasa and Wirata (45)/Indonesia | BECA | Prevention and early detection | Breast cancer | To provide information related to breast cancer awareness and facilitate BSE | - Personal data - Information about breast cancer - Information about breast self-examination - Notes on daily breast self-examination - Reminder feature that can be lit according to the user's time set | Android devices | Not reported | - Bethesda Yakkum Institute of Health Sciences | Not reported | Selected area of Yogyakarta |
| Zhu, Chen (39)/China | Breast Cancer e-Support (BCS) | Supportive and palliative care | Breast cancer | To support women with breast cancer undergoing chemotherapy | - Learning Forum - Discussion Forum - Ask-the-Expert Forum - Your Story Forum | Android and iOS devices | Bandura’s self-efficacy theory and social exchange theory | - Xiamen University, Xiamen, China - China-Japan Friendship Hospital, Beijing, China - University of Newcastle, Newcastle, Australia | Not reported | Patients from Zhong Shan Hospital and Hunan Cancer Hospital |
| Yaacob, Mohamad Marzuki (38)/Malaysia | ColorApp | Prevention and early detection | Colorectal cancer | To facilitate health education and promotion on colorectal cancer | - Introduction to colorectal cancer - Signs and symptoms of colorectal cancer - Risk factors of colorectal cancer - Preventions of colorectal cancer - Available screening programs in Malaysia - Immunochemical fecal occult blood test kits - Two interactive pages to stratify the user’s risk of disease - Health calculator that provides a recommendation on the need for:   - Screening   - Ideal body weight   - Recommended blood pressure, glucose, and cholesterol level - A video on colorectal cancer | Android devices | Not reported | - Universiti Sains Malaysia Health Campus, Kubang Kerian, Kelantan, Malaysia - Kedah State Health Department, Alor Setar, Kedah, Malaysia - Hospital Sultanah Bahiyah, Alor Setar, Kedah, Malaysia | Not reported | Kota Setar district |
| Wang, Chen (46)/China | Prostate cancer and clinicopathology risk calculator (PCCRC) | Screening and early detection | Prostate cancer | To monitoring the risk of prostate cancer and clinicopathology | - Age - Total prostate-specific antigen - Free prostate-specific antigen - Prostate volume - Free/total prostate-specific antigen - Prostate imaging reporting and data system version 2 - Probability of prostate cancer - Probability of Gleason score ≥ 7 - Probability of clinical stage ≥ T2b - Suggestions | NR | Not reported | - The First Affiliated Hospital of Anhui Medical University, Hefei, People’s Republic of China - Anhui Medical University, Hefei, People’s Republic of China - Anhui Medical University, Hefei, People’s Republic of China | Not reported | The First  Affiliated Hospital of Anhui Medical  University, Hefei |
| Rubagumya, Nyagabona (47)/Tanzania | NgoziYangu | Screening and early detection | Skin Cancer | To aid in the detection of skin cancer in people with albinism | - Patient information (e.g. sex, contacts, date of birth) - Acquire images either directly through the mobile camera or by uploading an image that had already been taken and stored - Skin lesion characteristics - Web portal for expert reviewers to view deidentified images and brief clinical information, and provide advice on management | Android devices and web portal | Not reported | - Rwanda Military Hospital, Kigali, Rwanda - University of Global Health Equity, Burera, Rwanda - Muhimbili University of Health and Allied Sciences, Dar es Salaam, Tanzania - Polyclinique du Mill ´enaire de Kisangani, Kisangani, Democratic Republic of Congo - Ocean Road Cancer Institute, Dar es Salaam, Tanzania - Regional Dermatology Training Centre, Moshi, Tanzania - Kilimanjaro Christian Medical Centre, Moshi, Tanzania - Inshuti Mubuzima, Kigali, Rwanda - Cancer Research Institute at Queen’s University, Kingston, Canada - Queen’s University, Kingston, Ontario, Canada | Not reported | Patients from Ocean Road Cancer Institute |
| Hou, Lan (56)/Taiwan | Breast Cancer Self-Management Support Mobile Health app | Supportive and palliative care | Breast cancer | To provide breast cancer self-management support in Taiwan | - Treatment (stages, treatment options, side effects, Chinese medicine care, meta and relapse, genetic, clinical trial) - Physical activity (exercise and rehabilitation) - Emotion (mental support, music therapy and good sleep) - Diet (diet guideline, receipts and food materials) - Health records (diet, emotion, physical activities, physical symptoms and social events) - Social resources (foundations, cancer centres, assistive technologies and economic support) - Experience sharing (sharing by illness stages, peer online gathering and supportive group) - Expert consulting (online consulting, instant messaging and frequently asked questions) | Android devices | Five steps design thinking model | - National Yang-Ming University, Taipei, Taiwan - National Taiwan University Hospital, Taipei, Taiwan - Taiwan Breast Cancer Foundation, Taipei, Taiwan - Brigham and Women’s Hospital, Boston, MA, United States - Harvard Medical School, Boston, MA, United States | Not reported | Patients from National Taiwan University Hospital, Taipei |
| Cheng, Ho (48)/China | Comprehensive intervention model supported by mHealth (CIMmH) | Supportive and palliative care | Esophageal cancer | To address poor nutrition, physical inactivity, and intensified mental health symptoms within a single program for patients with esophageal cancer after surgery | - General introduction - Nutrition guidelines - Physical exercise - Psychological support - Online support community | Not reported | Not reported | - The First Affiliated Hospital of Sun Yat-sen University, Guangzhou, China - The University of Hong Kong, Hong Kong, China - Sun-Yat-Sen University, Guangzhou, China - Sun Yat-sen Centre for Migrant Health Policy, Guangzhou, China - Sun Yat-sen Centre for Global Health, Guangzhou, China - Sun Yat-Sen University Cancer Centre, Guangzhou, China - University of Washington, Seattle, WA, United States - The University of Texas MD Anderson Cancer Centre, Houston, TX, United States | Not reported | Patients from the First Affiliated Hospital of Sun Yat-sen  University, Guangzhou |
| Marzuki, Yaacob (37)/Malaysia | ColorApp | Prevention and early detection | Colorectal cancer | To facilitate health education and promotion on colorectal cancer | - Introduction to colorectal cancer - Signs and symptoms of colorectal cancer - Risk factors of colorectal cancer - Preventions of colorectal cancer - Available screening programs in Malaysia - Immunochemical fecal occult blood test kits - Two interactive pages to stratify the user’s risk of disease - Health calculator that provides a recommendation on the need for:   - Screening   - Ideal body weight   - Recommended blood pressure, glucose and cholesterol level - A video on colorectal cancer | Android devices | Health belief model | - Universiti Sains Malaysia, Kota Bharu, Malaysia - Hospital Sultanah Bahiyah, Alor Setar, Malaysia - Kedah State Health Department, Alor Setar, Malaysia | Not reported | Kota Setar district in Kedah |
| Goulart Silveira, Carcano (49)/Brazil | Telederma app | Screening and early detection | Skin cancer | To take high quality digital images of suspicious lesions, capture patient data easily and quickly analyse skin cancer by healthcare professionals | - Registration of patients’ personal data - Clinical history and characterisation of the morphological features of each lesion - Acquire images through the mobile camera - Web-based portal for a dermatologist to provide diagnostic opinion on all lesions | Android devices and web portal | Not reported | - Barretos Cancer Hospital, Street Antenor Duarte Villela | Not reported | Individuals from the Cancer Prevention Department at Barretos Cancer Hospital |
| Zhu, Ebert (40)/China | Breast Cancer e-Support Program (BCS) | Supportive and palliative care | Breast cancer | To enhance women’s self-efficacy and social support, improve their symptom management, thus improving quality of life and psychological well-being | - Learning Forum - Discussion Forum - Ask-the-Expert Forum - Your Story Forum | Android and iOS devices | Bandura’s self-efficacy theory and social exchange theory | - Xiamen University, Xiamen, China - University of Newcastle, Newcastle, Australia | Not reported | Patients from Zhong Shan Hospital and Hunan Cancer Hospital |
| Quercia, Tran (50)/Madagascar | Cervical Cancer Prevention System (CCPS) | Screening and early detection | Cervical cancer | To monitor women participating in a cervical cancer screening campaign | - Screening information (time and place of the screening) - Administrative information (name, consent, photo, age, address, marital status, contact, employment status and education) - Obstetric history (gestity, parity, date of last periods, ongoing pregnancy, age of first sexual encounter, number of partners, contraception and duration) - Medical history (smoking, alcohol, drugs, HIV status, willing advice about HIV and HIV screening) - Cervical cancer history (knowledge of cervical cancer, familial history about cervical cancer and cervical cancer screening) - Screening (GeneXpert result, native colposcopy photo and result, photo with VIA and result and next appointment date) | Not reported | Not reported | - University of Geneva, Geneva, Switzerland - Geneva University Hospitals, Geneva, Switzerland - St-Damien Health Center, Ambanja, Madagascar - Geneva Foundation for Medical Education and Research, Geneva, Switzerland | Not reported | Saint Damien Healthcare  Center in Ambanja |
| Bhatt, Isaac (53)/India | SIM card application | Screening and early detection | Cervical and oral cancer | To support cancer screening activities in rural and remote communities | - Two separate forms for cervical cancer and oral cancer screening - A form for confirmation of treatment for both cancers - The screening form collected patient demographic details as well as a range of symptom-related questions for cervical cancer and behaviour-related questions for oral cancer - The other two forms recorded whether or not a second test, a biopsy and/or treatment was completed and included the results for each | Not reported | Not reported | - Medic Mobile, Mumbai, India - Christian Medical College, Vellore, India - Weill Cornell Medical College, New York, New York, USA - University of Edinburgh, Edinburgh, UK | Not reported | Christian Medical College Vellore’s Rural Unit  for Health and Social Affairs in Tamil Nadu; Padhar Hospital in Madhya Pradesh; and Christian Hospital Mungeli in Chhattisgarh |
| Ginsburg, Chowdhury (51)/Bangladesh | Mobile application | Screening and early detection | Breast cancer | To increase clinic attendance for breast symptoms in rural Bangladesh: | - Features not clearly described | Android devices | Not reported | - Women’s College Research Institute - University of Toronto, Toronto, Ontario, Canada - mPower Social Enterprises, Dhaka, Bangladesh - Amader Gram, Khulna, Bangladesh - International Breast Cancer Research Foundation, Madison, Wisconsin, USA | Not reported | Khulna Division |
