# Supplementary material for: Mobile-Based Application Interventions to Enhance Cancer Control and Care in Low- and Middle-Income Countries: A Systematic Review
Source: Int J Public Health. 2023 Dec 5;68:1606413. doi: 10.3389/ijph.2023.1606413 (PMC10732306; doi:10.3389/ijph.2023.1606413)
Supplement: Supplementary file 2 [file Table2.docx]

**Title:** Mobile-based application interventions to enhance cancer control and care in low- and middle-income countries: a systematic review

Supplementary Table 2: Ovid MEDLINE(R) and In-Process, In-Data-Review & Other Non-Indexed Citations and Daily

| 1 exp malignant neoplasm/ 3886085  2 (Neoplas* or Malignan* or Malignant Neoplasm or cancer* or Benign neoplasm or Tumo$r or Malignant tumo$r).ab,kw,ti. 3461783  3 1 or 2 4934862  4 exp mobile application/ 11764  5 (Mobile application* or mobile app* or portable software app* or portable software application* or tablet application).ab,kw,ti. 10366  6 4 or 5 17916  7 exp developing country/ 81294  8 (Countr* or under$developed countr* or developing area or developing countries or less$developed countr* or third$world country).ab,kw,ti. 595978  9 7 or 8 622985  10 exp Afghanistan/ 3823  11 Afghanistan.ab,kw,ti. 7063  12 10 or 11 8185  13  14 Burkina Faso.ab,kw,ti. 5127  15 13 or 14 5739  16 exp Burundi/ 718  17 Burundi.ab,kw,ti. 1049  18 16 or 17 1231  19 exp Central African Republic/ 835  20 Central African Republic.ab,kw,ti. 1170  21 19 or 20 1371  22 exp Chad/ 818  23 Chad.ab,kw,ti. 1510  24 22 or 23 1666  25 exp Democratic Republic Congo/ 0  26 Democratic Republic Congo.ab,kw,ti. 16  27 25 or 26 16  28 exp Eritrea/ 413  29 Eritrea.ab,kw,ti. 705  30 28 or 29 804  31 exp Ethiopia/ 19634  32 Ethiopia.ab,kw,ti. 28007  33 31 or 32 30719  34 exp Gambia/ 2712  35 Gambia.ab,kw,ti. 2772  36 34 or 35 3671  37 exp Guinea/ 1314  38 Guinea.ab,kw,ti. 109351  39 37 or 38 109591  40 exp Guinea-Bissau/ 1028  41 Guinea-Bissau.ab,kw,ti. 1131  42 40 or 41 1403  43 exp Korea/ 59356  44 Korea Democratic Republic.ab,kw,ti. 0  45 43 or 44 59356  46 exp Liberia/ 1397  47 Liberia.ab,kw,ti. 1936  48 46 or 47 2295  49 46 or 47 2295  50 exp Madagascar/ 3925  51 Madagascar.ab,kw,ti. 5752  52 50 or 51 5941  53 exp Malawi/ 6781  54 Malawi.ab,kw,ti. 8976  55 53 or 54 9868  56 exp Mali/ 2712  57 exp Mozambique/ 3055  58 Mozambique.ab,kw,ti. 4597  59 57 or 58 5006  60 57 or 58 5006  61 Mozambique.ab,kw,ti. 4601  62 Mozambique.ab,kw,ti. 4601  63 61 or 62 4601  64 exp Niger/ 1416  65 Niger.ab,kw,ti. 14618  66 64 or 65 14812  67 exp Rwanda/ 3175  68 Rwanda.ab,kw,ti. 4115  69 67 or 68 4756  70 exp Sierra Leone/ 1949  71 Sierra Leone.ab,kw,ti. 2894  72 70 or 71 3190  73 exp Somalia/ 1801  74 Somalia.ab,kw,ti. 1824  75 73 or 74 2869  76 exp South Sudan/ 268  77 South Sudan.ab,kw,ti. 788  78 76 or 77 811  79 exp Sudan/ 5184  80 Sudan.ab,kw,ti. 9678  81 79 or 80 11037  82 exp Togo/ 1278  83 Togo.ab,kw,ti. 1823  84 82 or 83 2037  85 exp Uganda/ 15552  86 Uganda.ab,kw,ti. 19216  87 85 or 86 21824  88 exp Yemen/ 1577  89 Yemen.ab,kw,ti. 2290  90 88 or 89 2718  91 exp Zambia/ 5577  92 Zambia.ab,kw,ti. 6779  93 91 or 92 7900  94 exp Angola/ 1139  95 Angola.ab,kw,ti. 1754  96 94 or 95 1981  97 exp Algeria/ 3559  98 Algeria.ab,kw,ti. 4243  99 97 or 98 5253  100 exp Bangladesh/ 14583  101 Bangladesh.ab,kw,ti. 19558  102 100 or 101 21861  103 exp Benin/ 1936  104 Benin.ab,kw,ti. 4136  105 103 or 104 4413  106 exp Bhutan/ 679  107 Bhutan.ab,kw,ti. 1102  108 106 or 107 1265  109 exp Bolivia/ 2843  110 Bolivia.ab,kw,ti. 3869  111 109 or 110 4708  112 exp Cape Verde/ 249  113 Cabo Verde.ab,kw,ti. 151  114 112 or 113 357  115 exp Cambodia/ 3875  116 Cambodia.ab,kw,ti. 4855  117 115 or 116 5819  118 exp Cameroon/ 6581  119 Cameroon.ab,kw,ti. 8645  120 118 or 119 9814  121 exp Comoros/ 373  122 Comoros.ab,kw,ti. 434  123 121 or 122 644  124 exp Congo/ 2015  125 Congo.ab,kw,ti. 15809  126 124 or 125 16453  127 exp Cote d'Ivoire/ 3585  128 Ivory Coast.ab,kw,ti. 1930  129 127 or 128 4285  130 exp Djibouti/ 244  131 Djibouti.ab,kw,ti. 444  132 130 or 131 501  133 exp Egypt/ 17658  134 Egypt.ab,kw,ti. 18670  135 133 or 134 26452  136 exp El Salvador/ 956  137 El Salvador.ab,kw,ti. 1493  138 136 or 137 1711  139 exp Eswatini/ 765  140 Eswatini.ab,kw,ti. 344  141 139 or 140 903  142 exp Ghana/ 11374  143 Ghana.ab,kw,ti. 15061  144 142 or 143 16770  145 exp Haiti/ 3601  146 Haiti.ab,kw,ti. 3570  147 145 or 146 4756  148 exp Honduras/ 1248  149 Honduras.ab,kw,ti. 2101  150 148 or 149 2389  151 exp India/ 119628  152 India.ab,kw,ti. 133315  153 151 or 152 183581  154 exp Indonesia/ 13740  155 Indonesia.ab,kw,ti. 18827  156 exp Kenya/ 19681  157 Kenya.ab,kw,ti. 22937  158 156 or 157 27262  159 154 or 155 22901  160 exp Kiribati/ 2151  161 Kiribati.ab,kw,ti. 248  162 160 or 161 2303  163 exp Kyrgyzstan/ 1392  164 Kyrgyz Republic.ab,kw,ti. 118  165 163 or 164 1442  166 exp Lebanon/ 5244  167 Lebanon.ab,kw,ti. 6301  168 166 or 167 7951  169 exp Lesotho/ 525  170 Lesotho.ab,kw,ti. 937  171 169 or 170 1005  172 exp Mauritania/ 491  173 Mauritania.ab,kw,ti. 758  174 172 or 173 858  175 exp "Federated States of Micronesia"/ 0  176 Micronesia.ab,kw,ti. 822  177 175 or 176 822  178 exp Mongolia/ 2100  179 Mongolia.ab,kw,ti. 5642  180 178 or 179 6279  181 exp Morocco/ 6607  182 Morocco.ab,kw,ti. 7461  183 181 or 182 9924  184 exp Myanmar/ 3217  185 Myanmar.ab,kw,ti. 4680  186 184 or 185 5625  187 exp Nepal/ 10805  188 Nepal.ab,kw,ti. 13525  189 187 or 188 15768  190 exp Nicaragua/ 1619  191 Nicaragua.ab,kw,ti. 2169  192 190 or 191 2507  193 Nigeria.mp. [mp=title, book title, abstract, original title, name of substance word, subject heading word, floating sub-heading word, keyword heading word, organism supplementary concept word, protocol supplementary concept word, rare disease supplementary concept word, unique identifier, synonyms, population supplementary concept word, anatomy supplementary concept word] 46240  194 Nigeria.ab,kw,ti. 36992  195 193 or 194 46240  196 Pakistan.mp. [mp=title, book title, abstract, original title, name of substance word, subject heading word, floating sub-heading word, keyword heading word, organism supplementary concept word, protocol supplementary concept word, rare disease supplementary concept word, unique identifier, synonyms, population supplementary concept word, anatomy supplementary concept word] 34576  197 Pakistan.ab,kw,ti. 27350  198 196 or 197 34576  199 Papua New Guinea.mp. [mp=title, book title, abstract, original title, name of substance word, subject heading word, floating sub-heading word, keyword heading word, organism supplementary concept word, protocol supplementary concept word, rare disease supplementary concept word, unique identifier, synonyms, population supplementary concept word, anatomy supplementary concept word] 5853  200 Papua New Guinea.ab,kw,ti. 4993  201 199 or 200 5853  202 Philippines.mp. [mp=title, book title, abstract, original title, name of substance word, subject heading word, floating sub-heading word, keyword heading word, organism supplementary concept word, protocol supplementary concept word, rare disease supplementary concept word, unique identifier, synonyms, population supplementary concept word, anatomy supplementary concept word] 14628  203 Philippines.ab,kw,ti. 11010  204 202 or 203 14628  205 Samoa.mp. [mp=title, book title, abstract, original title, name of substance word, subject heading word, floating sub-heading word, keyword heading word, organism supplementary concept word, protocol supplementary concept word, rare disease supplementary concept word, unique identifier, synonyms, population supplementary concept word, anatomy supplementary concept word] 1347  206 Samoa.ab,kw,ti. 1088  207 205 or 206 1347  208 exp Senegal/ 6251  209 Senegal.ab,kw,ti. 6650  210 208 or 209 8726  211 exp Solomon Islands/ 7154  212 Solomon Islands.ab,kw,ti. 958  213 211 or 212 7560  214 exp Tanzania/ 14001  215 Tanzania.ab,kw,ti. 16542  216 214 or 215 19247  217 exp Tajikistan/ 802  218 Tajikistan.ab,kw,ti. 761  219 217 or 218 1211  220 exp Timor-Leste/ 266  221 Timor-Leste.ab,kw,ti. 465  222 220 or 221 546  223 exp Tunisia/ 9277  224 Tunisia.ab,kw,ti. 8208  225 223 or 224 12101  226 exp Ukraine/ 17245  227 Ukraine.ab,kw. 5644  228 226 or 227 19663  229 exp Uzbekistan/ 1989  230 Uzbekistan.ab,kw,ti. 1428  231 229 or 230 2531  232 exp Vanuatu/ 416  233 Vanuatu.ab,kw,ti. 774  234 232 or 233 844  235 exp Viet Nam/ 14910  236 Vietnam.ab,kw,ti. 19113  237 235 or 236 23480  238 exp Zimbabwe/ 6604  239 Zimbabwe.ab,kw,ti. 6901  240 238 or 239 9026  241 exp Albania/ 954  242 Albania.ab,kw,ti. 1328  243 241 or 242 1630  244 exp American Samoa/ 201  245 American Samoa.ab,kw,ti. 412  246 244 or 245 446  247 exp Argentina/ 18031  248 Argentina.ab,kw,ti. 20555  249 247 or 248 26464  250 exp Armenia/ 1571  251 Armenia.ab,kw,ti. 1408  252 250 or 251 2242  253 exp Azerbaijan/ 1287  254 Azerbaijan.ab,kw,ti. 1721  255 253 or 254 2311  256 exp Belarus/ 2147  257 Belarus.ab,kw,ti. 1507  258 256 or 257 2941  259 exp Belize/ 641  260 Belize.ab,kw,ti. 941  261 259 or 260 1115  262 (Bosnia and Herzegovina).mp. [mp=title, book title, abstract, original title, name of substance word, subject heading word, floating sub-heading word, keyword heading word, organism supplementary concept word, protocol supplementary concept word, rare disease supplementary concept word, unique identifier, synonyms, population supplementary concept word, anatomy supplementary concept word] 3268  263 (Bosnia and Herzegovina).ab,kw,ti. 2117  264 262 or 263 3268  265 exp Botswana/ 2206  266 Botswana.ab,kw,ti. 3033  267 265 or 266 3373  268 exp Brazil/ 117686  269 Brazil.ab,kw,ti. 107226  270 268 or 269 152248  271 exp Bulgaria/ 6702  272 Bulgaria.ab,kw,ti. 5147  273 271 or 272 8941  274 exp China/ 275962  275 China.ab,kw,ti. 293766  276 274 or 275 407771  277 exp Colombia/ 13383  278 Colombia.ab,kw,ti. 16933  279 277 or 278 20351  280 exp Costa Rica/ 4041  281 Costa Rica.ab,kw,ti. 5805  282 280 or 281 6623  283 exp Cuba/ 5362  284 Cuba.ab,kw,ti. 5151  285 283 or 284 7472  286 exp Dominica/ 104  287 Dominica.ab,kw,ti. 571  288 286 or 287 592  289 exp Dominican Republic/ 1761  290 Dominican Republic.ab,kw,ti. 2354  291 289 or 290 2882  292 exp Equatorial Guinea/ 310  293 Equatorial Guinea.ab,kw,ti. 501  294 292 or 293 572  295 exp Ecuador/ 4701  296 Ecuador.ab,kw,ti. 6323  297 295 or 296 7681  298 exp Fiji/ 1129  299 Fiji.ab,kw,ti. 2340  300 298 or 299 2553  301 exp Gabon/ 1630  302 Gabon.ab,kw,ti. 2047  303 301 or 302 2443  304 exp "Georgia (republic)"/ 2002  305 Georgia.ab,kw,ti. 12000  306 304 or 305 12901  307 exp Grenada/ 162  308 Grenada.ab,kw,ti. 373  309 307 or 308 412  310 exp Guatemala/ 3337  311 Guatemala.ab,kw,ti. 4253  312 310 or 311 5173  313 exp Guyana/ 745  314 Guyana.ab,kw,ti. 1135  315 313 or 314 1442  316 exp Iraq/ 5571  317 Iraq.ab,kw,ti. 8642  318 316 or 317 10870  319 exp Jamaica/ 3616  320 Jamaica.ab,kw,ti. 3293  321 319 or 320 5033  322 exp Jordan/ 5438  323 Jordan.ab,kw,ti. 8711  324 322 or 323 9944  325 exp Kazakhstan/ 3121  326 Kazakhstan.ab,kw,ti. 3201  327 325 or 326 4600  328 exp Kosovo/ 301  329 Kosovo.ab,kw,ti. 1100  330 328 or 329 1143  331 exp Libyan Arab Jamahiriya/ 0  332 Libya.ab,kw,ti. 1567  333 331 or 332 1567  334 exp Malaysia/ 18124  335 Malaysia.ab,kw,ti. 21560  336 334 or 335 27892  337 exp Maldives/ 9  338 Maldives.ab,kw,ti. 501  339 337 or 338 501  340 exp Marshall Islands/ 2151  341 Marshall Islands.ab,kw,ti. 342  342 340 or 341 2316  343 exp Mauritius/ 619  344 Mauritius.ab,kw,ti. 1167  345 343 or 344 1282  346 exp Mexico/ 44220  347 Mexico.ab,kw,ti. 52266  348 346 or 347 68601  349 exp Moldova/ 743  350 Moldova.ab,kw,ti. 681  351 349 or 350 1146  352 exp "Montenegro (republic)"/ 0  353 Montenegro.ab,kw,ti. 1030  354 352 or 353 1030  355 exp Namibia/ 1315  356 Namibia.ab,kw,ti. 2051  357 355 or 356 2335  358 exp "Republic of North Macedonia"/ 639  359 North Macedonia.ab,kw,ti. 235  360 358 or 359 803  361 exp Palau/ 214  362 Palau.ab,kw,ti. 461  363 361 or 362 502  364 exp Paraguay/ 919  365 Paraguay.ab,kw,ti. 1887  366 364 or 365 2100  367 exp Peru/ 10875  368 Peru.ab,kw,ti. 13865  369 367 or 368 16955  370 exp Russian Federation/ 0  371 Russian Federation.ab,kw,ti. 4584  372 370 or 371 4584  373 exp Serbia/ 3781  374 Serbia.ab,kw,ti. 5591  375 373 or 374 6801  376 exp South Africa/ 49791  377 South Africa.ab,kw,ti. 44901  378 376 or 377 65866  379 exp Suriname/ 1012  380 Suriname.ab,kw,ti. 748  381 379 or 380 1388  382 exp Thailand/ 30963  383 Thailand.ab,kw,ti. 33756  384 382 or 383 44260  385 exp Tonga/ 279  386 Tonga.ab,kw,ti. 549  387 385 or 386 626  388 exp Turkmenistan/ 581  389 Turkmenistan.ab,kw,ti. 431  390 388 or 389 851  391 exp Tuvalu/ 2151  392 Tuvalu.ab,kw,ti. 84  393 391 or 392 2210  394 Mali.ab,kw,ti. 4378  395 56 or 394 4909  396 9 or 12 or 15 or 18 or 21 or 24 or 27 or 30 or 33 or 36 or 39 or 42 or 45 or 48 or 52 or 55 or 59 or 63 or 66 or 69 or 72 or 75 or 78 or 81 or 84 or 87 or 90 or 93 or 96 or 99 or 102 or 105 or 108 or 111 or 114 or 117 or 120 or 123 or 126 or 129 or 132 or 135 or 138 or 141 or 144 or 147 or 150 or 153 or 158 or 159 or 162 or 165 or 168 or 171 or 174 or 177 or 180 or 183 or 186 or 189 or 192 or 195 or 198 or 201 or 204 or 207 or 210 or 213 or 216 or 219 or 222 or 225 or 228 or 231 or 234 or 237 or 240 or 243 or 246 or 249 or 252 or 255 or 258 or 261 or 264 or 267 or 270 or 273 or 276 or 279 or 282 or 285 or 288 or 291 or 294 or 297 or 300 or 303 or 306 or 309 or 312 or 315 or 318 or 321 or 324 or 327 or 330 or 333 or 336 or 339 or 342 or 345 or 348 or 351 or 354 or 357 or 360 or 363 or 366 or 369 or 372 or 375 or 378 or 381 or 384 or 387 or 390 or 393 or 395 2118450  397 3 and 6 and 396 88 |
| --- |
