# Supplementary material for: Mobile-Based Application Interventions to Enhance Cancer Control and Care in Low- and Middle-Income Countries: A Systematic Review
Source: Int J Public Health. 2023 Dec 5;68:1606413. doi: 10.3389/ijph.2023.1606413 (PMC10732306; doi:10.3389/ijph.2023.1606413)
Supplement: Supplementary file 3 [file Table3.docx]

**Title:** Mobile-based application interventions to enhance cancer control and care in low- and middle-income countries: a systematic review

Supplementary Table 3: Critical assessment of the included studies (low- and middle-income countries, 2014 – 2022)

**Quasi-experiment studies**

| **JBI Checklist Questions** | **Wang et al. (2021)** | **Shakery et al. (2021)** | **Adiyasa and Wirata (2021)** | **Yaacob et al. (2020)** |
| --- | --- | --- | --- | --- |
| Is it clear in the study what is the ‘cause’ and what is the ‘effect’? | Yes | Yes | Yes | Yes |
| Were the participants included in any comparisons similar? | Yes | Yes | Yes | Yes |
| Were the participants included in any comparisons receiving similar treatment/care, other than the exposure or intervention of interest? | Yes | No | No | No |
| Was there a control group? | Yes | Yes | No | Yes |
| Were there multiple measurements of the outcome both pre and post the intervention/exposure? | Yes | Yes | No | No |
| Was follow up complete and if not, were differences between groups in terms of their follow up adequately described and analyzed? | Yes | Yes | Yes | Yes |
| Were the outcomes of participants included in any comparisons measured in the same way? | Yes | Yes | Yes | Yes |
| Were outcomes measured in a reliable way? | Yes | Yes | No | Yes |
| Was appropriate statistical analysis used? | Yes | Yes | No | Yes |

**Randomised controlled trials**

| **JBI Checklist Questions** | **Zhu et al. (2020)** | **Ginsburg et al. (2014)** |
| --- | --- | --- |
| Was true randomization used for assignment of participants to treatment groups? | Yes | Yes |
| Was allocation to treatment groups concealed? | NA | No |
| Were treatment groups similar at the baseline? | Yes | Yes |
| Were participants blind to treatment assignment? | No | No |
| Were those delivering treatment blind to treatment assignment? | No | No |
| Were outcomes assessors blind to treatment assignment? | No | No |
| Were treatment groups treated identically other than the intervention of interest? | Yes | Yes |
| Was follow up complete and if not, were differences between groups in terms of their follow up adequately described and analyzed? | Yes | Yes |
| Were participants analyzed in the groups to which they were randomized? | Yes | Yes |
| Were outcomes measured in the same way for treatment groups? | Yes | Yes |
| Were outcomes measured in a reliable way? | Yes | Yes |
| Was appropriate statistical analysis used? | Yes | Yes |
| Was the trial design appropriate, and any deviations from the standard RCT design (individual randomization, parallel groups) accounted for in the conduct and analysis of the trial? | Yes | Yes |

**Cross-sectional studies**

| **JBI Checklist Questions** | **Salmani et al. (2022)** | **Cavalcanti et al. (2021)** | **Quercia et al. (2018)** |
| --- | --- | --- | --- |
| Were the criteria for inclusion in the sample clearly defined? | Yes | Yes | Yes |
| Were the study subjects and the setting described in detail? | Yes | Yes | Yes |
| Was the exposure measured in a valid and reliable way? | Yes | Yes | Yes |
| Were objective, standard criteria used for measurement of the condition? | Yes | Yes | Yes |
| Were confounding factors identified? | No | No | No |
| Were strategies to deal with confounding factors stated? | No | No | No |
| Were the outcomes measured in a valid and reliable way? | Yes | Yes | No |
| Was appropriate statistical analysis used? | Yes | Yes | Yes |
|  |  |  |  |

**Qualitative study**

| **JBI Checklist Questions** | **Zhu et al. (2018)** |
| --- | --- |
| Is there congruity between the stated philosophical perspective and the research methodology? | Yes |
| Is there congruity between the research methodology and the research question or objectives? | Yes |
| Is there congruity between the research methodology and the methods used to collect data? | Yes |
| Is there congruity between the research methodology and the representation and analysis of data? | Yes |
| Is there congruity between the research methodology and the interpretation of results? | Yes |
| Is there a statement locating the researcher culturally or theoretically? | Yes |
| Is the influence of the researcher on the research, and vice- versa, addressed? | No |
| Are participants, and their voices, adequately represented? | Yes |
| Is the research ethical according to current criteria or, for recent studies, and is there evidence of ethical approval by an appropriate body? | No |
| Do the conclusions drawn in the research report flow from the analysis, or interpretation, of the data? | Yes |

**Mixed Methods**

| **Mixed Methods Appraisal Tool Questions** | **Rezaee et al. (2022)** | **Cavalcante Pires et al. (2022)** | **Ayyoubzadeh et al. (2022)** | **Hou et al. (2020)** | **Marzuki et al. (2019)** | **Bhatt et al. (2018)** |
| --- | --- | --- | --- | --- | --- | --- |
| Are there clear research questions? | Yes | No | Yes | Yes | Yes | Yes |
| Do the collected data allow to address the research questions? | Yes |  | Yes | Yes | Yes | Yes |
| Is there an adequate rationale for using a mixed methods design to address the research question? | Yes | No | Yes | Yes | Yes | Yes |
| Are the different components of the study effectively integrated to answer the research question? | Yes | No | No | Yes | Yes | Yes |
| Are the outputs of the integration of qualitative and quantitative components adequately interpreted? | No | No | No | Yes | No | No |
| Are divergences and inconsistencies between quantitative and qualitative results adequately addressed? | Yes | No | No | No | No | No |
| Do the different components of the study adhere to the quality criteria of each tradition of the methods involved? | No | No | No | Yes | No | No |

**Cohort studies**

| **CASP Checklist Questions** | **Rubagumya et al. (2020)** | **Cheng et al. (2020)** | **Goulart Silveira et al. (2019)** |
| --- | --- | --- | --- |
| Did the study address a clearly focused issue? | Yes | Yes | Yes |
| Was the cohort recruited in an acceptable way? | Yes | Yes | Yes |
| Was the exposure accurately measured to minimise bias | Yes | Yes | Yes |
| Was the outcome accurately measured to minimise bias | Yes | Yes | Yes |
| Have the authors identified all important confounding factors? | No | No | No |
| Have they taken account of the confounding factors in the design and/or analysis? | No | No | No |
| Was the follow up of subjects complete enough? | Yes | Yes | Yes |
| Was the follow up of subjects long enough? | Yes | Yes | Yes |
| Do you believe the results? | Yes | Yes | Yes |
| Can the results be applied to the local population? | No | Yes | No |
| Do the results of this study fit with other available evidence? | Yes | Yes | Yes |
